# Draft Genome Sequence of *Tepidiphilus thermophilus* Strain JHK30^T^ (JCM 19170^T^) Isolated from a Terrestrial Hot Spring in India

**DOI:** 10.1128/genomeA.00832-16

**Published:** 2016-08-11

**Authors:** Abhijit Poddar, Rinchen T. Lepcha, William B. Whitman, Subrata K. Das

**Affiliations:** a Department of Biotechnology, Institute of Life Sciences, Bhubaneswar, India; b Department of Microbiology, University of Georgia, Athens, Georgia, USA

## Abstract

*Tepidiphilus thermophilus* strain JHK30^T^ was isolated from a hot spring at Surajkund, Jharkhand, India. It is a Gram-negative rod, nonsporulating, aerobic, and motile. The estimated genome is 2.3 Mb, with 2,186 protein-coding sequences.

## GENOME ANNOUNCEMENT

*Tepidiphilus thermophilus* strain JHK30^T^ was isolated from sediment samples collected from a terrestrial hot spring at Surajkund, Jharkhand, India (1). This bacterium can grow at 30 to 60°C, and optimum growth occurs at 50 to 55°C. The *Tepidiphilus thermophilus* whole-genome sequence will help identify the genes encoding biotechnologically important enzymes.

The draft genome of *Tepidiphilus thermophilus* was generated using the Illumina HiSeq 2000 platform, which generated 8,078,364 paired-end reads with a total of 1,219.8 Mb and approximately 239.1-fold coverage. Filtered Illumina reads were assembled using Velvet (version 1.2.07) (2). One- to 3-kb simulated paired-end reads were created from Velvet contigs using wgsim (version 0.3.0) (https://github.com/lh3/wgsim). Illumina reads were assembled with simulated read pairs using Allpaths-LG (version r46652) (3).

The genome was annotated using the JGI Microbial Genome Annotation Pipeline (4). Genes were identified using Prodigal (5), followed by manual curation using GenePRIMP (6) for finished genomes and draft genomes with <20 scaffolds (>50 kb), containing 85.4% of the genome. The final assembly was based on 1,195.4 Mb of Illumina data and 239.1-fold input read coverage. The predicted protein-coding genes (CDSs) were translated and used to search the National Center for Biotechnology Information (NCBI) nonredundant, UniProt, TIGRFam, Pfam, KEGG, COG, and InterPro databases. The tRNAscan-SE tool (7) was used to find tRNA genes, whereas rRNA genes were found by searches against models of the rRNA genes built from SILVA (8). Other noncoding RNAs, such as the RNA components of the protein secretion complex and the RNase P, were identified by searching the genome for the corresponding Rfam profiles using INFERNAL (http://infernal.janelia.org.).

The draft genome assembly of *Tepidiphilus thermophilus* contained 37 contigs in 35 scaffolds, with a total genome size of 2.3 Mb and *N*_50_ contig size of 141.2 kb. The largest contig was 363.6 kb long, with a G+C content of 66.1%. The draft genome sequence has 2,186 candidate protein-coding genes (CDSs) in addition to 68 RNAs, of which 46 were tRNAs and seven were rRNAs.

### Accession number(s).

This whole-genome shotgun project has been deposited at DDBJ/EMBL/GenBank under the accession no. LIPU00000000. The version described in this paper is the first version, LIPU01000000.
